# Supplementary material for: Graphene and Reproduction: A Love-Hate Relationship
Source: Nanomaterials (Basel). 2021 Feb 22;11(2):547. doi: 10.3390/nano11020547 (PMC7926437; doi:10.3390/nano11020547)
Supplement: Supplementary file 1 [file nanomaterials-11-00547-s001.zip › Supplementary Table 1.docx]

| **Supplementary Table 1.** |  |  |
| --- | --- | --- |
| **Main topological parameters computed on Co-authors networks** | |  |
|  |  |  |
| **Parameter** | **Network** | |
| Number of nodes | 176 |  |
| Number of edges | 776 |  |
| Clustering coefficient | 0,946 |  |
| Connected components | 20 |  |
| Network diameter | 3 |  |
| Shortest paths | 1964(6%) | |
| Characteristic path length | 1,272 |  |
| Avg. number of neighbours | 8,330 |  |
| **Node degree Distribution** |  |  |
| γ | -0,534 |  |
| r | 0,214 |  |
| R2 | 0,16 |  |
|  |  |  |
|  |  |  |
|  |  |  |
