# Supplementary material for: Graphene and Reproduction: A Love-Hate Relationship
Source: Nanomaterials (Basel). 2021 Feb 22;11(2):547. doi: 10.3390/nano11020547 (PMC7926437; doi:10.3390/nano11020547)
Supplement: Supplementary file 1 [file nanomaterials-11-00547-s001.zip › Supplementary Table 2.docx]

| **Authors** | **Journal** | **Year** | **Animal species** | **In vivo/ In vitro** | **Graphene compound** | **Doses** | **Toxicologic study?** | **Detrimental effects?** | **Additional Informantion /comments** |
| --- | --- | --- | --- | --- | --- | --- | --- | --- | --- |
| Zanni et al. | Nano Letters | 2012 | *C. Elegans* | In vivo | GNPs | 50, 100, 250 µg/mL | Yes | NO | Longevity and reproductive capability |
| Chatterjee et al. | Environ. Health and Toxicol. | 2015 | *C. Elegans* | In vivo | GO: SLGO, FLGO ; GNPs: GNPs-pristine, GNPs-COOH, GNPs-NH2 | 5, 10, 20, 50 µg/mL | Yes | Only with highest concentrations |  |
| Chatterjee et al. | Nanotoxicology | 2016 | *C. Elegans* | In vivo | GO and rGO | 20, 50, 100 µg/mL | Yes | Yes, higher for GO |  |
| Zhao et al. | Biomaterials | 2016 | *C. Elegans* | In vivo | GO | 1, 10, 100, 1000 µg/mL | Yes | Yes | Statistical analysis wrong? |
| Zhao et al. | Toxicology Research | 2015 | *C. Elegans* | In vivo | nitrogen-doped GQDs | 0.1, 1, 10, 100 µg/mL | Yes | NO |  |
| Kong et al. | Solid State Phenomena | 2019 | *C. Elegans* | In vivo | PLA-graphene | 50, 200, 500, 1000 µg/mL | Yes | NO | 48h |
| Dziewiecka et al. | Science of the Total Environ | 2018 | *A. Domesticus* | In vivo | GO | 20, 200 µg/g | Yes | Yes | Food |
| Mendonça et al. | Nanomaterials | 2019 | *E. crypticus* | In vivo | GO, rGO | 250,1000 mg/Kg | Yes | Yes | Food |
| Martins et al. | Chemosphere | 2019 | *S. frugiperda* | In vivo | 1D-oxidized MWCNT, 2D GO | 10, 100, 1000 µg/g | Yes | Yes | Food |
| Xu et al. | Biomaterials | 2015 | *Mouse* | In vivo | rGO (small and large) | 6.25, 12.5, 25 mg/kg | Yes | Depending on the injection days | IV injected, systemic evaluation, female mouse reproductive and offspring |
| Zhang et al. | Biomaterials | 2019 | *Mouse* | In vivo | Cu+2-labeled GQDs | 60, 100, 300 mg/Kg o.g, 25, 75, 150 mg/Kg IV | Yes | Yes | Oral gabage or IV injected |
| Park et al. | Nanotoxicology | 2016 | *Mouse* | In vivo | R and P-SWCNT | 50, 100 µg/kg | Yes | Yes | Intratracheally instilled, inflammatory response (Th1) |
| Liu et al. | Biomed Environ Sci | 2014 | *Zebrafish* | In vivo | MWCNTs, GO, rGO | 1, 5, 10, 50, 100 µg/mL | Yes | Yes | Developing embryos / Statistical analysis wrong? |
| Kim et al. | Toxicology | 2018 | *C. elegans* | In vivo | GO | 10 µg/mL | Yes | Yes | Spermatogenesis, accumulation in organs |
| Mesaric et al. | Aquatic Toxicology | 2015 | *P. lividus* | In vivo | CB, GO | 0.0001, 0.001, 0.01, 0.1, 1 µg/mL | Yes | Only CB | Sperm evaluation |
| Liang et al. | Nanotoxicology | 2014 | *Mouse* | In vivo | S-GO, L-GO | 120, 300 mg/kg S-GO -120 mg/kg L-GO | Yes | NO | sperm evaluation, IV injected tail vein |
| Akhavan et al. | Carbon | 2015 | *Mouse* | In vivo | NGO | 2, 20, 200, 2000 µg/mL | Yes | Yes | IV injected, confirmed uptake by the testis |
| Nirmal et al. | Bas Clin Pharmacol Toxicol | 2017 | *Wistar Rats* | In vivo | NGO | 0.4, 2 and 10 mg/kg | Yes | Only higher doses | Injected |
| Bernabo et al. | Carbon | 2018 | *Swine* | In vitro | GO | 0.5, 1, 5, 10, 50 µg/mL | Yes-NO | Only higher concentrations | IVF positive results |
| Bernabo et al. | Scientific Reports | 2019 | *Swine* | In vitro | GO | 0.5, 1, 1.5, 2.5, 5 µg/mL | NO | NO | cholesterol extraction |
| Ramal-Sanchez et al. | Carbon | 2019 | *Bovine* | In vitro | GO | 1 µg/mL | NO | NO | proteomic, lipidomic |
| Lin et al. | J Cellular Physiology | 2018 | *Mouse* | In vitro | GQDs | 0.5, 1, 1.5 µg/mL | Yes | Yes | Oocytes |
| Hashemi et al. | RSC Advances | 2014 | *Mouse* | In vitro | GO and rGO (N2H4-GO, HT-GO, GTPs-GO) | 0.1, 1, 10, 100, 400 µg/mL | Yes | highest concentrations | Sperm incubation |
| Asghar et al. | Scientific Reports | 2016 | *Human* | In vitro | SWCNT-COOH and rGO | 1, 5, 25 µg/mL | Yes | NO |  |
| Ema et al. | Nanotoxicology | 2015 |  |  |  |  |  |  | Review |
| Sajjad et al. | Chemistry Select | 2019 | *E. Coli, S. Aureus* | In vitro | GO/Ag2O NPs | Particle size from 36.3 to 46.4 nm | NO | Antibacterial activity | Agar disc diffusion method |
| Karpeta-Kaczmarek et al. | Environ. Research | 2018 | *A. Domesticus* | In vivo | Nanodiamonds | 20 and 200 μg/g | YES | Yes (high concentrations) | Food |
| Cao et al. | Int. J of Electrochemical Sci | 2017 | *E. Coli* | In vitro | Electrochemically reduced GO |  | NO | No | Improvement of bacterial reproduction |
| Das et al. | Human Reproduction Update | 2016 |  |  |  |  |  |  | Review |
| Wang et al. | Int J of Nanomedicine | 2018 |  |  |  |  |  |  | Review |
| Skovmand et al. | Particle and Fiber Toxicology | 2018 | *Mouse* | In vivo | GO, Flammruss 101, Printex 90, SRM1650b | 2 mg/ml | SI | Only in bronchoalveolar fluid, not in sperm parameters | Particles dispersed in water |
| Bernabo et al. | Front. Bioeng. Biotechnol | 2020 | *Mouse* | In vitro | GO | 0.1, 0.5, 1, 5, 10, 50 µg/mL | NO | Only high concentrations | IVF, embryo development: positive results. Healthy offspring obtained |
| Jha et al. | Mat Sci and Engineering C | 2016 | Human | In vitro | Fe3O4, MWCNT and graphene platelet nanopowder (GPN) |  | NO | Only Fe3O4 and GPNs | MWCNT do not disturb the sperm lipid membrane |
